# To the Editors of the Medical and Physical Journal

**Published:** 1802-07

**Authors:** Thomas Beddoes


### *To the Editors of the Medical and Phyſical Journal.*

GENTLEMEN,

I Was one of thoſe whom an apparently unfavourable caſe led at firſt to doubt of the ſecurity afforded by the cow-pox againſt the ſmall-pox. However, on account of my very doubts, I attended with deeper intereſt to the inveſtigation; and it is now long ſince I offered the poor of Briſtol the benefit of the new Inoculation at the Pneumatic Inſtitution. Every inſtance has confirmed the good effect of the practice. The deviations from the uſual appearances, Mr. King will relate in the account of our tranſactions.

With as high a ſenſe of what mankind owe to Dr. Jenner as has been expreſſed by any of your correſpondents, I cannot but be deeply mortified at the ſmallneſs of the parliamentary reward which he is likely to receive. The largeſt ſum now propoſed muſt, I think, be felt as very inadequate; and without a NATIONAL SUBSCRIPTION, the communication of diſcoveries of immediate and general utility, will be checked. I feel this more ſenſibly, as I ſhall have to announce in my eighth Eſſay on Health, a diſcovery of apparently very great importance, but of which the author, not being a medical man, can receive no emolument but through the means of ſecrecy, public remuneration, or private contribution.

I hope to ſee by the newſpapers, or your next number, that many others feel as I do. If profeſſional men exert themſelves, they will find abundance of families, who have received, or hope to receive, benefit from Dr. Jenner's labours, ready to help towards advancing his fortune; and he ſurely will not bluſh to receive ſuch proofs of their ſenſe of obligation. Probably thoſe very Members of Parliament, who from a ſenſe of duty, ſhewed themſelves moſt ſparing of the public purſe, will be among the moſt forward to open their own.

I am, &c.

*June* 4, 1802. THOMAS BEDDOES.

